# The molecular mechanism of G_2_/M cell cycle arrest induced by AFB_1_ in the jejunum

**DOI:** 10.18632/oncotarget.9594

**Published:** 2016-05-25

**Authors:** Heng Yin, Min Jiang, Xi Peng, Hengmin Cui, Yi Zhou, Min He, Zhicai Zuo, Ping Ouyang, Junde Fan, Jing Fang

**Affiliations:** ^1^ Key Laboratory of Animal Diseases and Environmental Hazards of Sichuan Province, Chengdu, Sichuan, PR China; ^2^ College of Veterinary Medicine, Sichuan Agricultural University, Chengdu, Sichuan, PR China; ^3^ College of Biological and Agro-Forestry Engineering, Tongren University, Tongren, Guizhou, PR China

**Keywords:** AFB_1_, G_2_/M phase, cell cycle arrest, mechanism, jejunum, Pathology Section

## Abstract

Aflatoxin B_1_ (AFB_1_) has potent hepatotoxic, carcinogenic, genotoxic, immunotoxic and other adverse effects in human and animals. The aim of this study was to investigate the molecular mechanism of G_2_/M cell cycle arrest induced by AFB_1_ in the jejunum of broilers. Broilers, as experimental animals, were fed 0.6 mg/kg AFB_1_ diet for 3 weeks. Our results showed that AFB_1_ reduced the jejunal villus height, villus height/crypt ratio and caused G_2_/M cell cycle arrest. The G_2_/M cell cycle was accompanied by the increase of ataxia telangiectasia mutated (ATM), p53, Chk2, p21 protein and mRNA expression, and the decrease of Mdm2, cdc25C, cdc2, cyclin B and proliferating cell nuclear antigen protein and mRNA expression. In conclusion, AFB_1_ blocked G_2_/M cell cycle by ATM pathway in the jejunum of broilers.

## INTRODUCTION

Aflatoxins, secondary metabolites produced in feedstuffs by *Aspergillus flavus* and *Aspergillus parasiticus*, which cause health and economic problems when they contaminate food and feed. These mycotoxins are difuranocoumarin compounds and include B_1_, B_2_, G_1_, G_2_, M_1_ and M_2_ [1]. Of these toxins, aflatoxin B_1_ (AFB_1_) is the most commonly encountered and it is considered to have higher toxicity than other aflatoxins. AFB_1_ also has potent hepatotoxic, genotoxic, immunotoxic and other adverse effects in many animal species including humans [1, 2–4].

The eukaryotic cell cycle is central to maintain homeostasis in the multicellular organisms [5, 6]. In response to various types of DNA damages, the cell cycle regulatory molecules and cell death signals are activated to stop cell growth and to eliminate multiplication of genetically altered cells [7, 8]. AFB_1_ could cause significant increase of S-phase cell population in murine macrophages and human bronchial epithelial cells *in vitro* [9, 10]. In murine models, a significantly greater proportion of lung cells were found to enter cell cycle with extended S-phase due to AFB_1_ treatment [11]. Scott et al. [2] have demonstrated that AFB_1_ treatment can lead to an accumulation of chicken thymocytes in G_2_/M phase *in vitro*. Our previous studies suggest that AFB_1_ induced jejunal cell arrest at G_2_/M phase and renal cell arrest at G_0_/G_1_ phase in broilers [12, 13].

The small intestine is the primary digestive apparatus of animals, and AFB_1_ transfer across the gastrointestinal wall is an apparently important step in the fate of the toxin in the animal body [14, 15]. As part of the small intestine, the jejunum is the major component of the gastrointestinal tract. Although there are some studies on cell cycle arrest induced by AFB_1_, few reports focus on the relationship between AFB_1_ and cell cycle in the jejunum, and the exact mechanism of G_2_/M cell cycle arrest induced by AFB_1_ in jejunum has not been elucidated. To address this, we used a broiler model to examine the effects of dietary AFB_1_ in jejunum. We analyzed the histological changes of jejunum, the cell cycle, and the protein and mRNA expression levels of regulatory molecules involved in G_2_/M transition. The results could provide helpful information for the further studies in human and other animals in the future.

## RESULTS

### Pathological observation of jejunum

**Figure 1:**
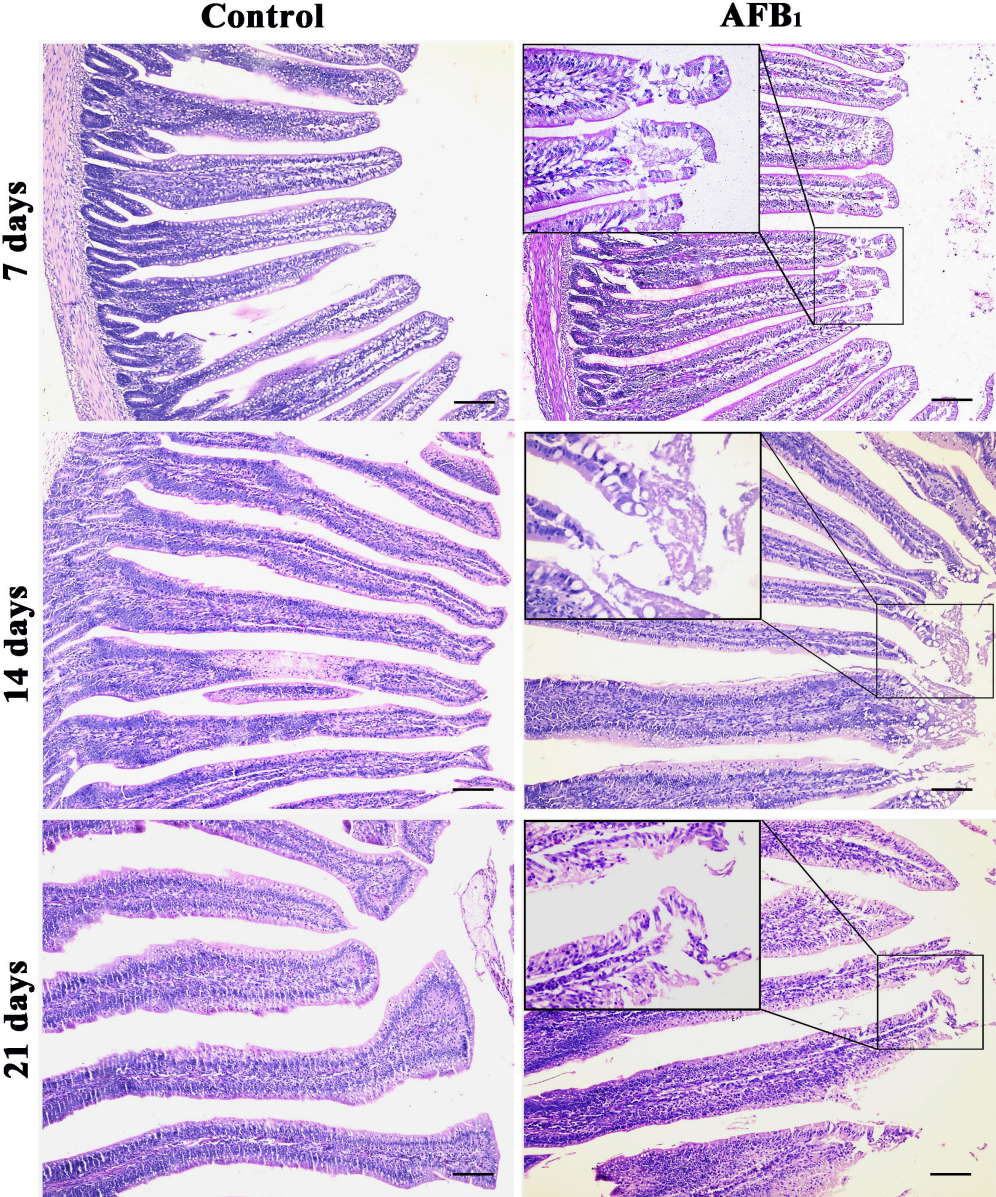
Histological structures of jejunum at 7, 14 and 21 days of age (H.E staining, scale bar: 100 μm)

Compared with the control group, the epithelial cells in the apical region of jejunal villus were shedding in the AFB_1_ group at 7, 14 and 21 days of age (Figure 1).

### Villus height, crypt depth and villus/crypt ratio in the jejunum

**Figure 2:**
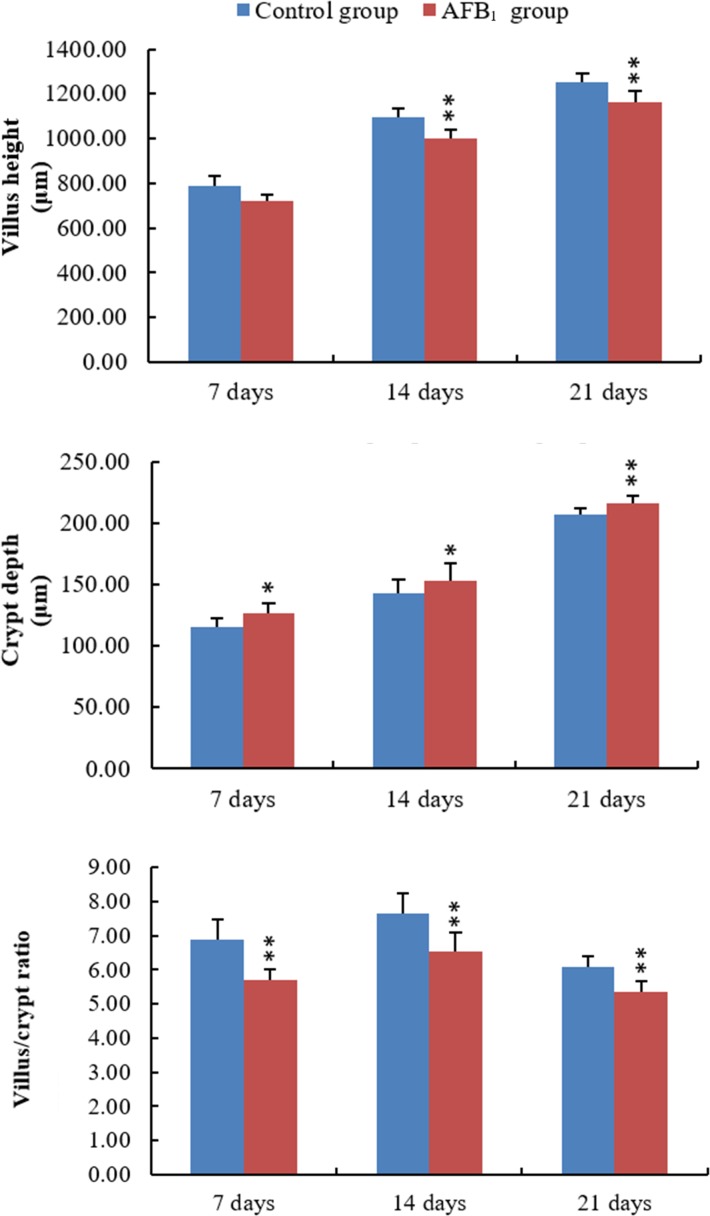
The values of villus height, crypt depth and villus/crypt ratio in the jejunum Note: data are presented with the means ± standard deviation (*n* = 6).*p < 0.05, **p < 0.01 compared with control group.

The villus heights in the AFB_1_ group were significantly lower than those in the control group at 14 and 21 days of age (*p* < 0.01), with the exception of villus height on day 7 which did not show a significant decrease in comparison to that in the control group (*p* > 0.05). The crypt depth in the AFB_1_ group significantly increased at 7, 14 and 21 days of age (*p* < 0.05 or 0.01), when compared with that in the control group. In addition, the villus/crypt ratios in the AFB_1_ group were significantly lower than those in the control group at 7, 14 and 21 days of age (*p* < 0.01) (Figure 2).

### Cell cycle in the jejunal mucosa

**Figure 3:**
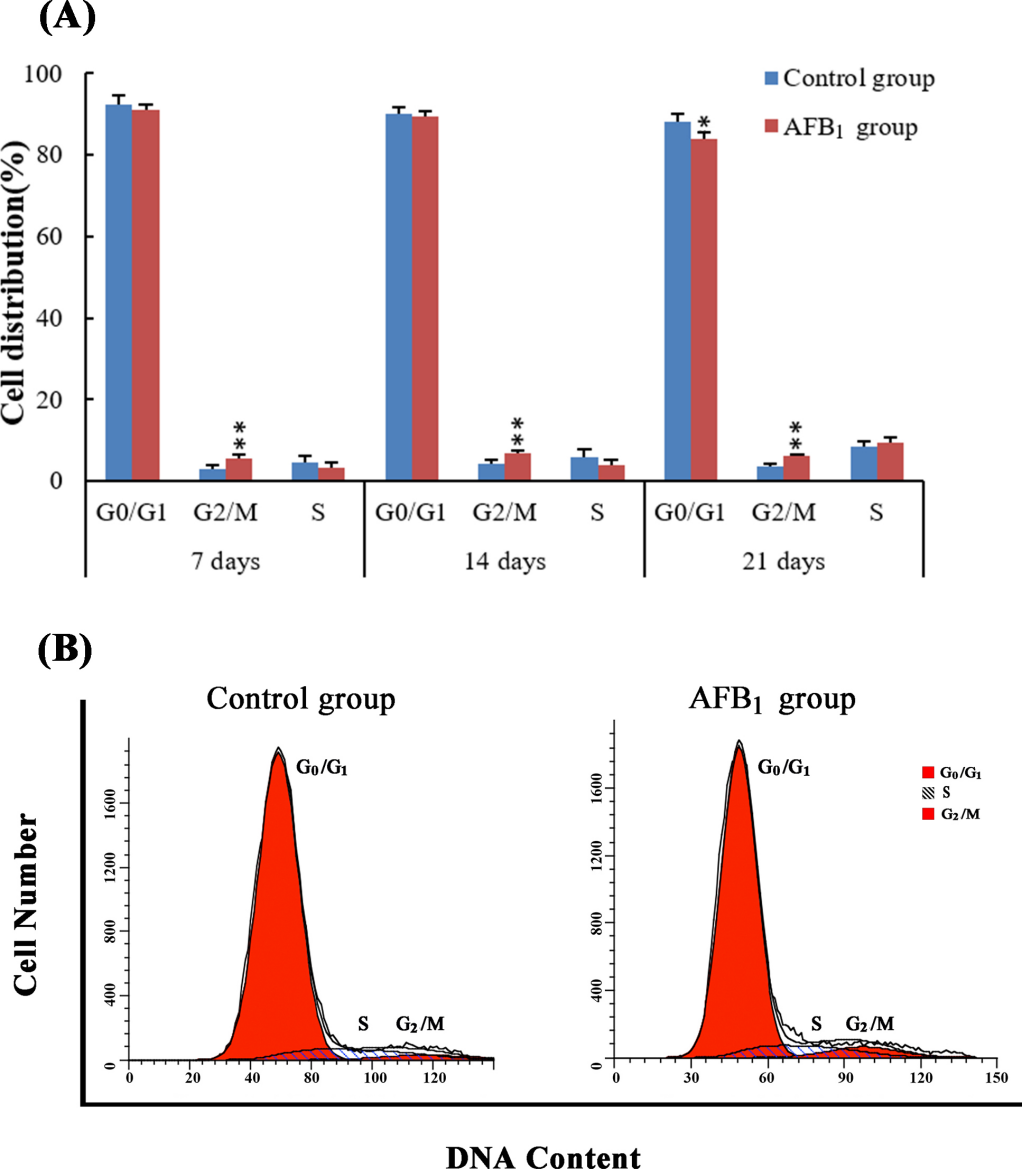
The cell cycle of jejunal mucosa by flow cytometry **Panel A**: Percentage of cells in G_0_/G_1_, S and G_2_/M phases. Data are presented with the means ± standard deviation (*n* = 6) *p < 0.05, **p < 0.01 compared with control group. **Panel B**: Flow cytometric analysis of cell cycle distribution in the jejunal mucosa cells at 14 days of age, showing cell cycle arrest in G_2_/M phase induced by AFB_1_.

The percentage of cells in G_0_/G_1_ phase was significantly lower in the AFB_1_ group than that in the control group at 21 days of age(*p* < 0.05), but the change was not obvious at 7 and 14 days of age (*p* > 0.05). The cell percentages of G_2_/M phase were significantly higher in the AFB_1_ group than those in the control group at 7, 14 and 21 days of age (*p* < 0.01). No significant differences in cell percentage of S phase were noted between two groups at 7, 14 and 21 days (*p* > 0.05) (Figure 3).

### Expression of cell cycle regulatory molecule proteins by immunohistochemistry

**Figure 4:**
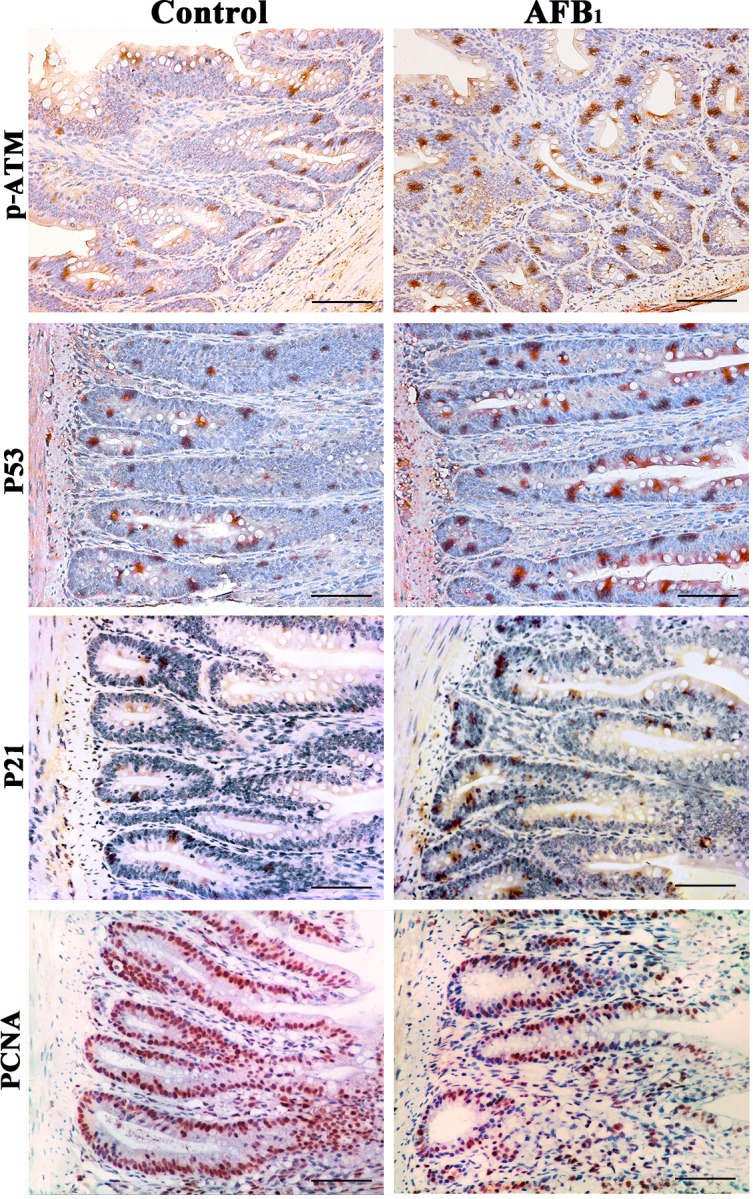
Expression of p-ATM, p53, p21 and PCNA protein in the jejunum at 14 days of age (Immunohistochemistry, scale bar: 50μm)

**Figure 5:**
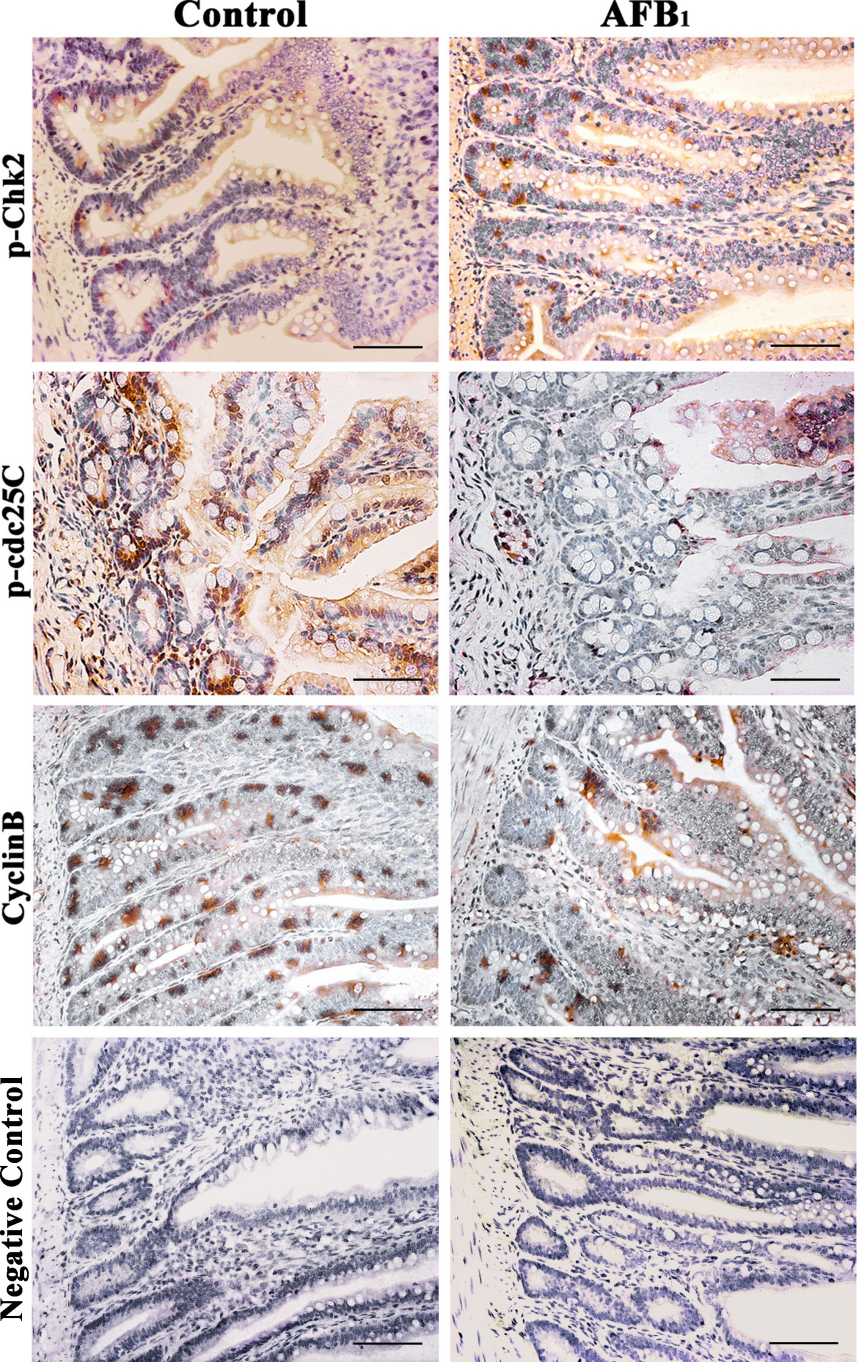
Expression of p-Chk2, p-cdc25C and cyclinB protein in the jejunum at 14 days of age (Immunohistochemistry, scale bar: 50μm)

**Figure 6:**
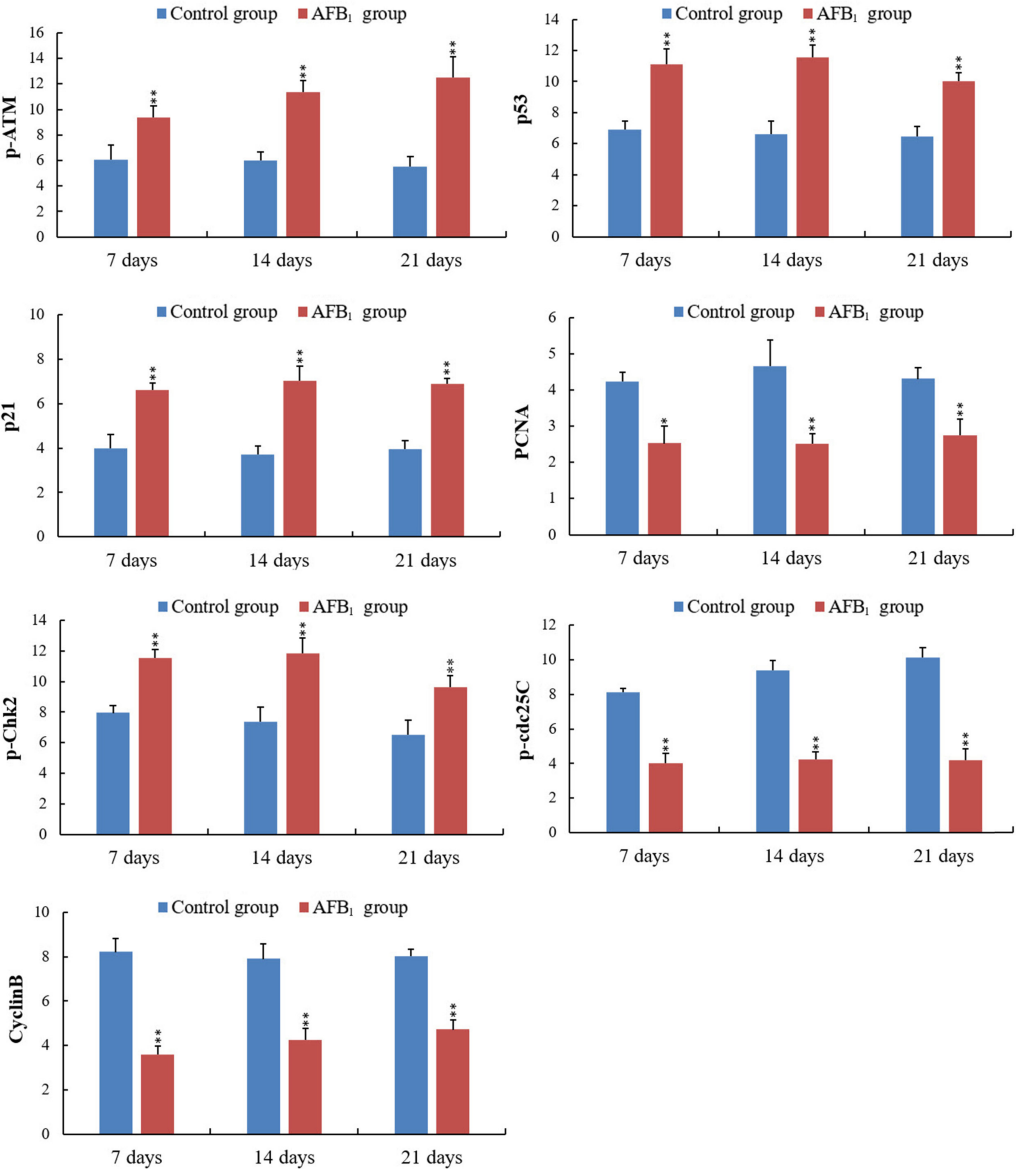
Integrated optical density (IOD) of p-ATM, p53, p21, PCNA, p-Chk2, p-cdc25C and CyclinB protein expression in the jejunum Note: Data are presented with the mean ± standard deviation (*n* = 6).*p < 0.05, **p < 0.01 compared with the control group.

Stained brownish-yellow, the expression of G_2_/M cell cycle regulatory molecule proteins is shown in Figure 4, 5 and 6. Control sections showed negative reaction (Figure 5).

The p-ATM protein expression was significantly increased (*p* < 0.01) in the AFB_1_ group at 7, 14 and 21 days of age, when compared with those in the control group. The p53 and p21 protein expression were significantly higher (*p* < 0.01) in the AFB_1_ group at 7, 14 and 21 days of age than those in the control group. But compared with the control group, the PCNA protein expression in the AFB_1_ group was significantly decreased at 7, 14 and 21 days of age (*p* < 0.05 or 0.01) (Figure 6).

The p-Chk2 protein expression was significantly higher (*p* < 0.01) in the AFB_1_ group than that in the control group from 7 to 21 days of age. The p-cdc25C and cyclin B protein expression were significantly decreased (*p* < 0.01) in the AFB_1_ group at 7, 14 and 21 days of age compared with those in the control group (Figure 6).

### The expression of cell cycle regulatory mRNA by qRT-PCR

**Figure 7:**
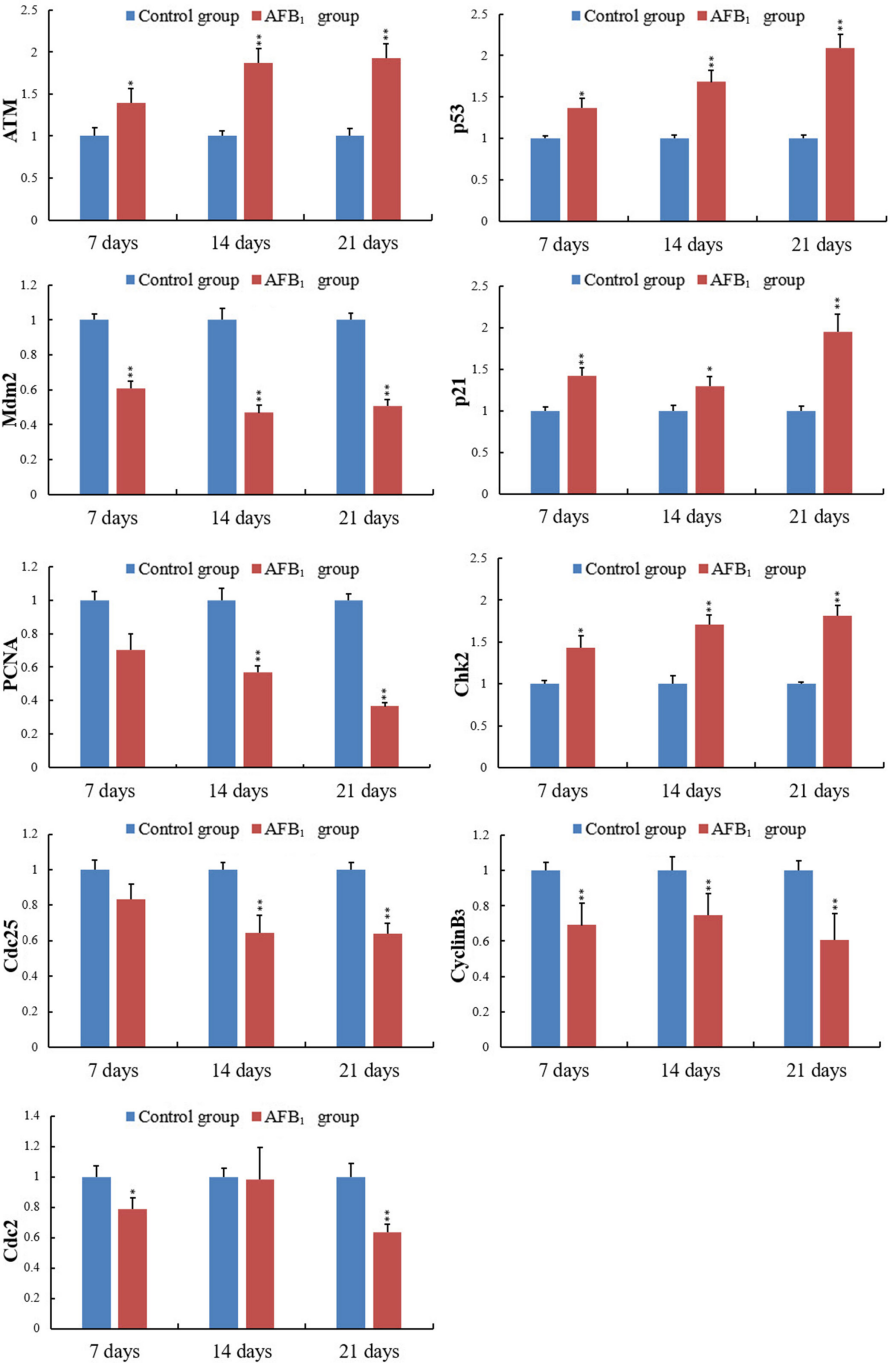
The levels of the ATM, Chk2, cdc25, CyclinB3, cdc2, mdm2, p53, p21 and PCNA mRNA expression in the jejunal mucosa Note: Data are presented with the mean ± standard deviation (*n* = 6).*p < 0.05, **p < 0.01 compared with the control group.

When compared with the control group, the mRNA expression levels of ATM, Chk2 and CyclinB_3_ in the AFB_1_ group were obviously increased at 7,14 and 21 days of age (*p* < 0.05 or 0.01). The mRNA expression of cdc25 was decreased at 14 and 21 days of age (*p* < 0.01), but not significantly dropped at 7 days of age (*p* > 0.5). And the expression levels of cdc2 mRNA in the AFB_1_ group were obviously decreased at 7 and 21 days of age (*p* < 0.05 or 0.01), but not significantly changed at 14 days of age compared with the control group (*p* > 0.5) (Figure 7).

The mRNA expression of p53 and p21 was obviously increased at 7, 14 and 21 days of age after exposure to AFB_1_ (*p* < 0.05 or 0.01). The mRNA expression of Mdm2 and PCNA became significantly decreased at 7, 14 and 21 days of age compared with those of the control group (*p* < 0.01) except for PCNA (Figure 7).

## DISCUSSION

According to previous [12] and present researches, AFB_1_ retarded the development of jejunum, including the shedding of villus and the reduction of the villus height and villus height/crypt ratio, which may be dependent on the decreased abilities of cell proliferation. As we know, the shedding of villus should be responsible for lower villus height. However, significant lower villus heights were observed in the AFB_1_ group at 14 and 21 days and not at 7 days in this study. The reason for this may be complicated because villus growth involves epithelial cell proliferation rates, migration rates of epithelial cells from the crypts to the villus tip, or apoptosis and exfoliation rates of epithelial cells [16]. Therefore, besides shedding, other factors may also be responsible for lower villus height. In addition, the morphological modifications reported in Figure 1 were that the epithelial cells in the apical region of jejunal villus were shedding. As reported in Figure 3 lower percentage of cells in G_0_/G_1_ is observed only at 21 days and is only minor in the AFB_1_ group. This means that cell proliferation is lightly increased. The possible relevance of these changes to the morphological modifications reported in Figure 1 is that the enhancement of cell proliferation might be attributed to the repair ability due to the continuous damage to the epithelial cells which is the protective mechanism of the organism. But the key finding is that AFB_1_ induced jejunal cell cycle arrest at the G_2_/M phase, which was in line with our earlier research [12]. However, the mechanism of G_2_/M cell cycle arrest induced by AFB_1_ is unclear.

In this present study, the G_2_/M phase cell cycle regulatory molecules were measured in order to define the molecular mechanism of AFB_1_-induced G_2_/M phase arrest in broiler's jejunum. The ataxia telangiectasia mutated (ATM) plays a critical role in the activation of cell cycle checkpoints [17]. The ATM pathway responds mainly to DNA double strand breaks but also to other types of DNA damage [18]. The central position of ATM in the maintenance of genomic stability becomes apparent by its involvement in checkpoint regulation at the G_2_/M transition [19, 20]. At the DNA-damage, ATM activates Chk2 by phosphorylation [21], and then the Chk2 influences the stabilization of p53 [22]. Chk2 can induce G_2_/M cell cycle arrest through p53 up-regulation [22, 23]. Furthermore, AFB_1_ is metabolized to aflatoxin-8, 9-epoxide by cytochrome P450 (CYP450) microsomal enzymes, and the reactive aflatoxin-8, 9-epoxide can cause DNA damage [24]. In the present study, our results showed that AFB_1_ caused the increase of ATM, p-Chk2 and p53 protein and mRNA expression, suggesting that AFB_1_ activated the ATM signal transduction pathways by up-regulation of ATM, Chk2 and p53. Therefore, AFB_1_ induced G_2_/M cell cycle arrest *via* two different routes: the ATM-p53 and the ATM-Chk2 pathways. Similarly, Yang et al. [10] reported that ATM, ATR, Chk2 and p53 were up-regulated by AFB_1_ in B-2A13 cells. Gursoy-Yuzugullu et al. [25] found that AFB_1_ induced accumulation of p53 in HepG2 hepatoma cells.

P53 is a crucial mediator of at least two or more cellular responses to a variety of DNA damage: apoptosis, DNA repair and cell cycle arrest [26]. In normal conditions, p53 levels are low due to continuous Mdm2-mediated ubiquitination and degradation, and the activation of Mdm2 by p53 is required to reverse the inhibitory effects of p53 on cell cycle progression [27]. P53 can induce p21 up-regulation, which causes cells to arrest in G_2_/M phase [28]. Furthermore, p21 induces cell cycle arrest at G_2_/M phase by inhibiting cdc2 [29, 30]. An inactive cdc2/cyclin B complex does not allow cells to progress beyond the G_2_/M cell-cycle checkpoint. Meanwhile, p21 inhibits DNA replication and maintains G_2_/M cell cycle arrest through the binding to PCNA and the reduction of PCNA [31–33]. The PCNA, as a marker of proliferation, takes part in DNA biological synthesis and regulates cell cycle [34, 35]. The limitation of cdc2/cyclin B complex formation and the down-regulation of PCNA expression block the passage of cells to mitosis [31, 36]. In this study, our results showed that AFB_1_ caused the increase of p53 and p21 proteins and mRNA expression, and decrease of cyclin B and PCNA protein and mRNA expression as well as cdc2 and Mdm2 mRNA expression. Therefore, we speculated that dietary AFB_1_ could cause G_2_/M cell cycle arrest in jejunum through p53-dependent p21 activation. Similar results were also observed in the following references. β-Mangostin can induce the p53-dependent G_2_/M cell cycle arrest by down-regulating cdc2 and PCNA [37]. Exposure of HEK 293 cells to citrinin induced cell cycle G_2_/M arrest and increased the expression of p53 and p21 proteins [38]. Deoxynivalenol arrested epithelial cell cycle at G_2_/M phase *via* elevated p21 gene expression [39]. In human liver cells, Ochratoxin A (OTA) exerted a major influence on G_2_ phase arrest, in which the cyclin B1-cdc2 complex was reduced and the expression of cdc2 and cyclin B1 were significantly decreased by OTA treatment both at protein and mRNA level [40].

Herein, up-regulation of Chk2 protein and mRNA expression indicates that AFB_1_ activates ATM-Chk2 pathways to inhibit cdc2/cyclin B expression. The cyclin B/cdc2 complexes are also activated in prophase by the cdc25 phosphatase, while inhibition of cdc25 by the checkpoint kinases Chk2 prevents cyclin B/cdc2 activation [41, 42]. And the relatively low amounts of the formation of an active cyclin B/Cdc2 complex may be the main factor of cells accumulated in G_2_/M phase [43]. Our results showed that AFB_1_ caused the decrease of cdc25C expression, indicating that AFB_1_ induced G_2_/M phase arrest *via* ATM-Chk2-cdc25-cyclin B/cdc2 route.

**Figure 8:**
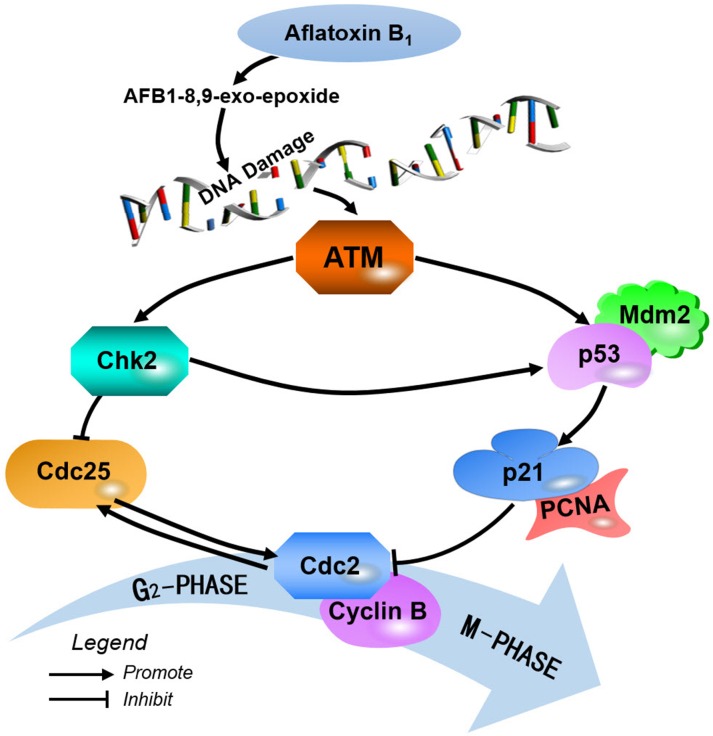
Schematic diagram of the proposed the molecular mechanism of aflatoxin B1 arrest G2/M cell cycle in broiler's jejunum

In summary, 0.6 mg/kg AFB_1_ in the diet inhibited the development of broiler's jejunum by causing G_2_/M cell cycle arrest. This was accompanied by the increase of ATM, p53, p21 and Chk2 proteins and mRNA expression, and the decrease of cdc25, cdc2, cyclin B, Mdm2 and PCNA proteins and mRNA expression. Here, we showed the proposed mechanisms of jejunal G_2_/M cell cycle arrest caused by AFB_1_ (Figure 8).

## MATERIALS AND METHODS

### Experimental design

One hundred and fifty-six one-day-old healthy Cobb broilers were randomly divided into two groups with three replicates per group and 26 birds per replicate, namely control group (0 mg/kg AFB_1_) and AFB_1_ group (0.6 mg/kg AFB_1_). The use of broilers and all experimental procedures involving animals were approved by Sichuan Agricultural University Animal Care and Use Committee. Nutritional requirements were adequate according to National Research Council (1994) (National Research Council, 1994) [44] and Chinese Feeding Standard of Chicken (NY/T33-2004).AFB_1_ was obtained from Sigma-Aldrich (USA, A6636). The AFB_1_-contaminated diet was made, similarly to the method described by Kaoud [45]. Briefly, 27 mg AFB_1_ farinose solid was dissolved into 30 mL methanol completely, and then the 30 mL mixture was mixed into 45 kg corn-soybean basal diet to formulate AFB_1_ diet of experimental groups containing 0.6mg/kg AFB_1_. The equivalent methanol was mixed into corn-soybean basal diet to produce control diet. Then, the methanol of diets was evaporated at 98 °F (37°C). The AFB_1_ concentrations were analyzed by HPLC (Waters, Milford, MA, USA) with fluorescence detection (Waters, Model 2475, Milford, MA, USA), and the AFB_1_ concentrations were determined as < 0.01mg/kg and 0.061mg/kg in the control diet and AFB_1_ diet, respectively. Broilers were housed in cages with electrically heated units and provided with water as well as above mentioned diet *ad libitum* for 21 days.

### Histopathological examination

At the end of 7, 14 and 21 days of experiment, six chickens in each group were euthanized, and jejunum (the midpoint between the bile duct entry and Meckel's diverticulum) were immediately fixed in 4% paraformaldehyde. After fixation for 24 h, tissues were dehydrated, paraffin embedded, sectioned at 5 μm, and stained with haematoxylin and eosin (H.E) for histological examination. Paraffin sections were also collected to perform immunohistochemistry. The histological structures of the tissues were observed and photographed with a digital camera (Nikon DS-Ri1, Japan). Altogether 10 measurements were taken per broiler for each parameter in the jejunum stained with H.E using Image-Pro Plus 5.1 (USA) image analysis software. The following parameters were determined: the villus height (length from the tip of the villus to the crypt mouth), crypt depth (length from the crypt mouth to the crypt base following the crypt lumen) and villus height to crypt depth (villus/crypt) ratio.

### Cell cycle analysis by flow cytometry

At the end of 7, 14 and 21 days, six chickens in each group were selected for the determination of the cell cycle stages in jejunum by flow cytometry, with a similar method described by Chen et al. [46]. Briefly, the chickens were humanely killed and the jejunal mucosae were obtained, and minced using scissors to form a cell suspension that was filtered through a 300-mesh nylon screen. The cells were washed twice with ice-cold phosphate buffer saline (PBS, pH 7.2-7.4), and then suspended in PBS at a concentration of 1 × 10^6^ cells/mL. A total of 500 μL of the cell suspension was transferred to a 5-mL culture tube. After centrifugation (200×g, 5 min), the supernatant was separated, the cells were incubated for 30 min at room temperature in the dark with 5 μL 0.25% Triton X-100 and 5 μL Propidium Iodide (PI) (Cat. No.51-66211E). Finally, 500 μL of PBS were added to each tube, and cells were analyzed by flow cytometry (BD FACSCalibur) within 45 min of preparation. The results of cell cycle were analyzed using the Mod Fit LT for Mac V3.0 computer program.

### Immunohistochemistry

The method of immunohistochemistry was applied according to the report by Fang et al. [47]. The jejunal paraffin sections were dewaxed in xylene, rehydrated through a graded series of ethanol solutions, washed in distilled water and PBS and endogenous peroxidase activity was blocked by incubation with 3% H_2_O_2_ in methanol for 15 min. The sections were saturated with normal 10% goat sera for 30 min in order to eliminate non specific irrelevant proteins staining and then incubated with the primary(rabbit/mouse)antibodies (Table 1) for 17 h at 4°C (working dilution: 1:100). After washing in PBS, the slices were exposed to 1% biotinylated goat anti-rabbit/mouse IgG secondary antibody (Boster, Wuhang, China) for 1 h at 37°C, and then incubated with strept avidin-biotin complex (SABC; Boster, Wuhang, China) for 30 min at 37°C. To visualize the immunoreaction, sections were immersed in diaminobenzidine hydrochloride (DAB; Boster, Wuhang, China). The slices were monitored microscopically and stopped by immersion in distilled water, as soon as brown staining was visible. Slices were lightly counterstained with hematoxylin, dehydrated in ethanol, cleared in xylene and mounted. For negative control purposes, representative sections were processed in the same way by replacing primary antibodies by PBS.

**Table 1:** Antibodies used in immunohistochemistry

Name	Company	Cat#	Source	Dilution
p-ATM	Bioss, China	bs-2272R	Rabbit	1:100
p-Chk2	Bioss, China	bs-3721R	Rabbit	1:100
p53	Boster, China	BM0101	Mouse	1:100
p21	Boster, China	BA0272	Rabbit	1:100
p-cdc25C	Bioss, China	bs-3482R	Rabbit	1:100
Cyclin B1	Boster, China	BA0766	Rabbit	1:100
PCNA	Bioss, China	bs-0754R	Rabbit	1:100

In this study, the protein expression levels of the cell cycle regulatory molecules were determined by the integrated optical density (IOD). Briefly, photographs of the jejunums were taken with a digital microscope camera system (Nikon DS-Ri1, Japan). For each section, five fields of 0.064 mm^2^ from each area of the image were analyzed using computer-assisted image-Pro Plus 5.1 (USA) image analysis software. By selecting “colour-chosen target” in the option bar of the morphologic analysis system, all positive immunoreactive cells in the field were marked in colour. Then, “calculating” in the option bar was selected to automatically calculate the IOD value.

### Quantitative real-time PCR

The jejunal mucosae from six chickens in each group were taken at 7, 14and 21 days of age and stored in liquid nitrogen. The jejunal mucosae were crushed with pestle to homogenize until powdery, respectively. As previously described [48], total RNA was extracted from the powdery of jejunal mucosae using RNAiso Plus (9108/9109, Takara, Otsu, Japan). Next, cDNA was synthesized using a Prim-Script™ RT reagent Kit (RR047A, Takara, Japan) according to the manufacture's protocol. The cDNA product was used as a template for qRT-PCR analysis. Sequences for target genes were obtained from the NCBI database. Oligonucleotide primers were designed using Primer 5 software and synthesized at Takara (Dalian, China; Table 2). All qRT-PCR were performed using the SYBR^®^ Premix Ex TaqTM II system (DRR820A, Takara, Japan) using on a Model C1000 Thermal Cycler (Bio Rad, USA). Chicken β-actin expression was used as an internal reference housekeeping gene. Gene expression values from control group subsamples at 7, 14, and 21 days of age were used to calibrate gene expression in subsamples from corresponding experimental subsamples. All data output from the qRT-PCR experiments were analyzed using the 2^−ΔΔCT^ method [49].

**Table 2:** Sequence of primers used in qRT-PCR

Gene symbol	Accession number	Primer	Primer sequence(5′-3′)	Product size	Tm (°C)
ATM	NM001162400.1	Forward Reverse	TTGCCACACTCTTTCCATGT CCCACTGCATATTCCTCCAT	110bp	60
Chk2	NM001080107	Forward Reverse	AGACCAAATCACTCGTGGAGAATAC GATGCTCTAAGGCTTCCTCTATTGT	140bp	60
cdc25	NM001199572.1	Forward Reverse	AGCGAAGATGATGACGGATT GCAGAGATGAAGAGCCAAAGA	163bp	59
p53	NM205264.1	Forward Reverse	ACCTGCACTTACTCCCCGGT TCTTATAGACGGCCACGGCG	127bp	59
Mdm2	AF005045.1	Forward Reverse	AACTTCCCAGCCAACAACA TCAAAGGTCAACGAGATGCT	123bp	59
p21	AF513031.1	Forward Reverse	TCCCTGCCCTGTACTGTCTAA GCGTGGGCTCTTCCTATACAT	123bp	60
cdc2	NM205314.1	Forward Reverse	TCTGCTCTGTATTCCACTCCTG ATTGTTGGGTGTCCCTAAAGC	144bp	60
cyclinB3	NM205239.2	Forward Reverse	ATCACCAACGCTCACAAGAAC AGGCTCCACAGGAACATCTG	171bp	59
PCNA	AB053163.1	Forward Reverse	GATGTTCCTCTCGTTGTGGAG CAGTGCAGTTAAGAGCCTTCC	104bp	60
β-actin	L08165	Forward Reverse	TGCTGTGTTCCCATCTATCG TTGGTGACAATACCGTGTTCA	178bp	62

### Statistical analysis

The significance of difference between two groups was analyzed by variance analysis, and results were expressed as mean ± standard deviation $\left(\bar{X}\pm \text{SD}\right)$. The analysis was performed using the independent sample test of SPSS 20.0 software (IBM Corp, Armonk, NY, USA) for windows. Statistical significance was considered at *p* < 0.05 and markedly significant was considered at *p* < 0.01.
